# Correction: QuEChERS pretreatment combined with high-performance liquid chromatography-tandem mass spectrometry for determination of aristolochic acids I and II in Chinese herbal patent medicines

**DOI:** 10.1039/d0ra90099k

**Published:** 2020-09-30

**Authors:** Jinghe Zhang, Yinan Wang, Jing Sun, Guowei Zhou, Xiaojie Jiang, Xikui Wang

**Affiliations:** Key Laboratory of Fine Chemicals in Universities of Shandong, School of Chemistry and Pharmaceutical Engineering, Qilu University of Technology (Shandong Academy of Sciences) Jinan 250100 China wangyn@qlu.edu.cn +86-531-89631696; School of Environmental Science and Engineering, Qilu University of Technology (Shandong Academy of Sciences) Jinan 250353 China; School of Environmental Science and Engineering, Shandong Agriculture and Engineering University Jinan 250100 China

## Abstract

Correction for ‘QuEChERS pretreatment combined with high-performance liquid chromatography-tandem mass spectrometry for determination of aristolochic acids I and II in Chinese herbal patent medicines’ by Jinghe Zhang *et al.*, *RSC Adv.*, 2020, **10**, 25319–25324, DOI: 10.1039/D0RA03200J.

The authors regret multiple errors in the original article. The corrections are listed below.

Firstly, the authors have amended their Graphical Abstract in order to further differentiate it from the cover image associated with Yinan Wang’s *Journal of Agricultural and Food Chemistry* paper, ‘Quantitation of Aristolochic Acids in Corn, Wheat Grain, and Soil Samples Collected in Serbia: Identifying a Novel Exposure Pathway in the Etiology of Balkan Endemic Nephropathy’ (DOI: 10.1021/acs.jafc.6b02203).

The authors regret that Fig. 2a in the original article showed the wrong spectrum. The corrected version of Fig. 2 is shown below with the correct spectra and removal of the *y*-axes labels.

**Fig. 2:**
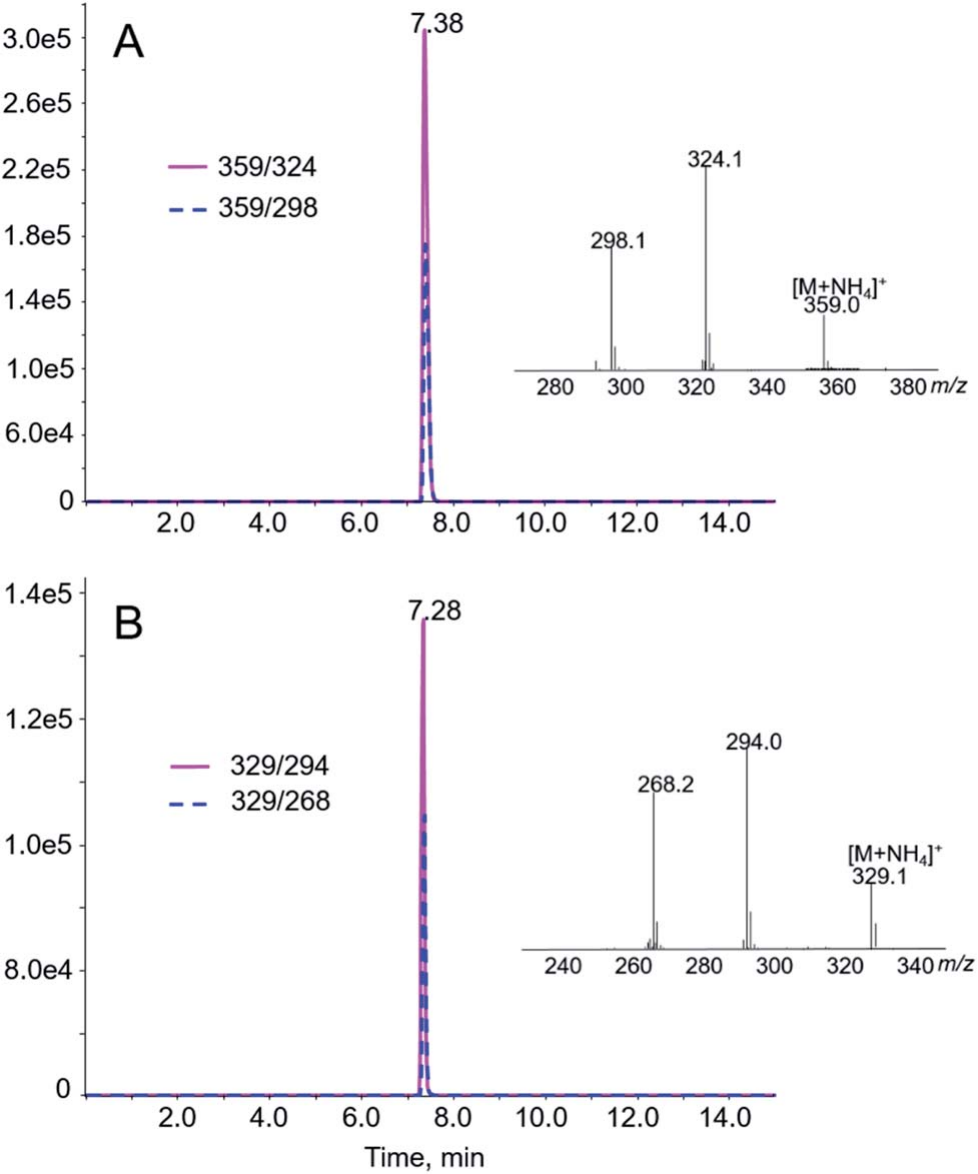
LC-MS/MS analyses of AA I (A) and AA II (B) in prepared standard solutions. AA I and AA II were eluted at 7.38 and 7.28 min, respectively. Shown in the insets are the product ion spectra of the [M + NH_4_]^+^ ion of AA I (A, *m*/*z* 359) and AA II (B, *m*/*z* 329).

The corrected version of Fig. 3 is also shown below with removal of the *y*-axes labels.

**Fig. 3:**
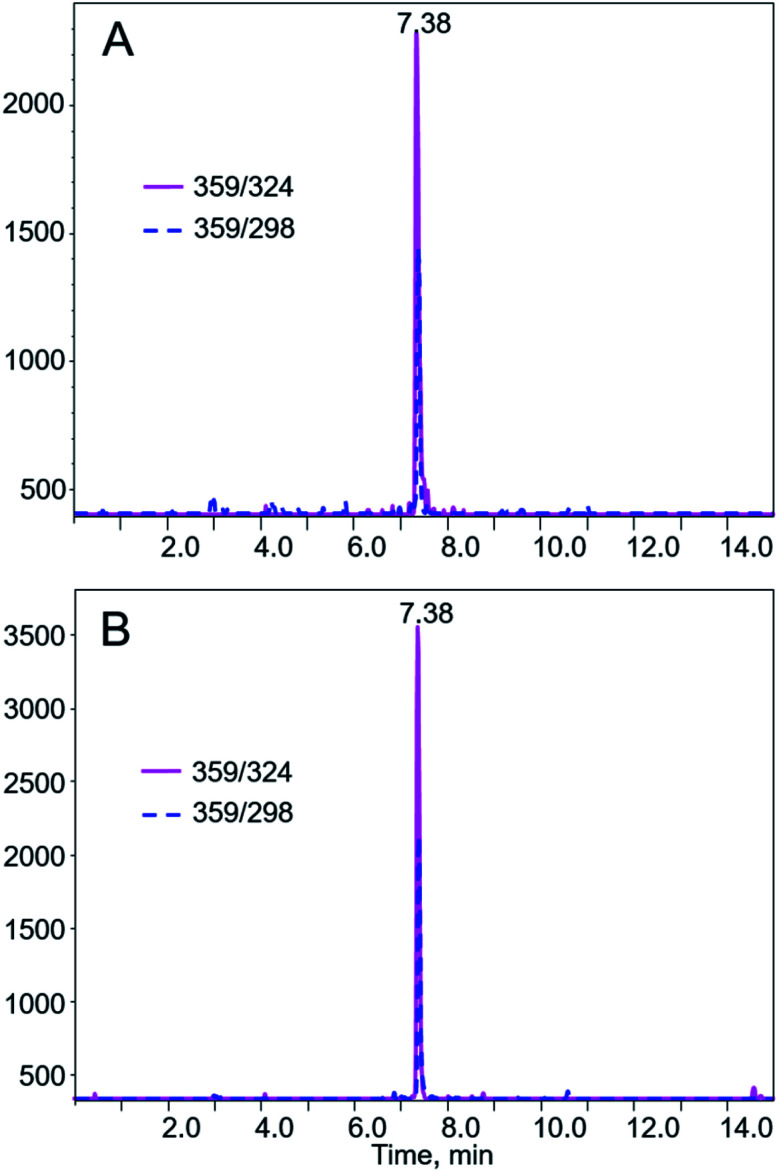
Typical LC-MS/MS chromatograms from MRM of AA I (*m*/*z* 359 → 324 and 359 → 298) in (A) liquid sample and (B) solid sample purchased from Alibaba of China. AA I was eluted at 7.38 min under the chromatographic condition described in the Materials and methods section. AA II is with concentration below the method detection limit.

The authors wish to remove Table 1 from the original article as this table contains instrumental parameters which do not correspond to the experimental conditions used in this work and was included in error.

The statement on line 8 of Section 2.3 on page 25320 ‘and 13–18 min, 10% (B, v/v).’ should be replaced with ‘and 13–15 min, 10% (B, v/v).’

The sentence starting on line 12 of Section 2.3 on page 25320 ‘The *m*/*z* values for MRM transitions for the target analytes and internal standard are listed in Table 1, where the residence time for each transition is set to 100 ms.’ should be replaced with ‘The MS/MS spectra of AA I and AA II were obtained using Agilent 1200RRLC-6520 Accurate-Mass Q-TOF LC/MS.’

The sentence starting on line 5 of Section 2.5 on page 25321 ‘Method precision was evaluated by analyzing herbal extracts spiked with AAs at two different concentrations (50 and 500 ng g^−1^) on the same day (*n* = 5) and over separate days in a month (*n* = 5).’ should be replaced with ‘Method precision was evaluated by analyzing herbal extracts spiked with AAs at two different concentrations (50 and 500 ng g^−1^) on the same day (*n* = 5) and over separate days in two weeks (*n* = 5).’

The first sentence of Section 3 on page 25321 ‘The extraction of AAs from each sample was conducted with methanol under sonication for 30 min, followed by d-SPE clean-up to isolate AAs from the extraction system.’ should be replaced by ‘The extraction of AAs from each sample was conducted with acetonitrile under sonication for 30 min, followed by d-SPE clean-up to isolate AAs from the extraction system.’

The sentence starting on line 19 of column 2 on page 25321 ‘Meanwhile, method precision was investigated by analysing blank samples spiked with AAs at two different concentrations (50 and 500 ng g^−1^) on the same day (*n* = 5) and over seven separate days for two weeks.’ should be replaced by ‘Meanwhile, method precision was investigated by analysing blank samples spiked with AAs at two different concentrations (50 and 500 ng g^−1^) on the same day (*n* = 5) and over five separate days for two weeks.’

The Royal Society of Chemistry apologises for these errors and any consequent inconvenience to authors and readers.

## Supplementary Material

